# Supplementary material for: A Novel CXCR4-Targeted Diphtheria Toxin Nanoparticle Inhibits Invasion and Metastatic Dissemination in a Head and Neck Squamous Cell Carcinoma Mouse Model
Source: Pharmaceutics. 2022 Apr 18;14(4):887. doi: 10.3390/pharmaceutics14040887 (PMC9032726; doi:10.3390/pharmaceutics14040887)
Supplement: Supplementary file 1 [file pharmaceutics-14-00887-s001.zip › pharmaceutics-1660429-supplementary.pdf]

pharmaceutics

Supplementary Materials: A Novel CXCR4-Targeted Diphtheria Toxin Nanoparticle Inhibits Invasion and Metastatic Dissemination in a Head-and-Neck Squamous-Cell-Carcinoma Mouse Model

Elisa Rioja-Blanco , Alberto Gallardo, Irene Arroyo-Solera, Patricia Álamo, Isolda Casanova, Ugutz Unzueta, Naroa Serna, Laura Sánchez-García, Miquel Quer, Antonio Villaverde, Esther Vázquez, Xavier León, Lorena Alba-Castellón and Ramon Mangues

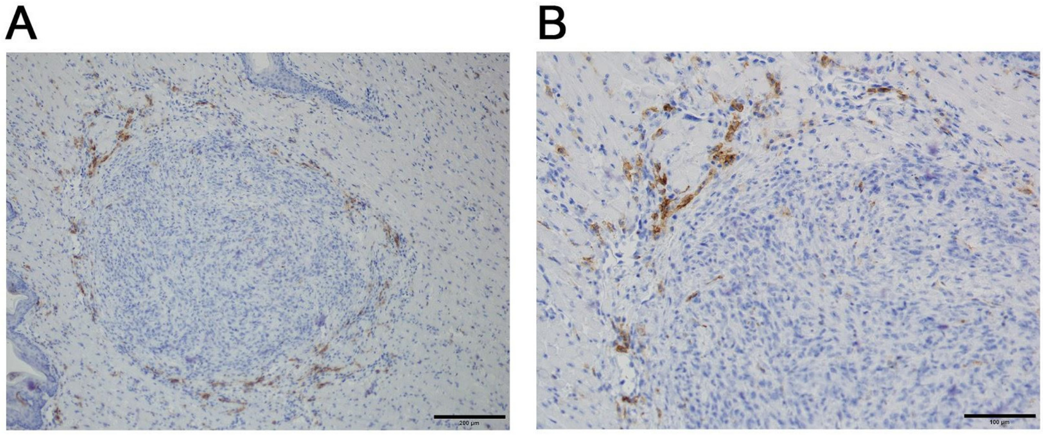

Figure S1. CXCR4 expression in 74B-Luci primary tumor samples in the orthotopic mouse model. CXCR4 IHC representative images of a 74B-Luci orthotopic tumor. **A**) Overall expression of CXCR4 within the primary tumor and in the tumor margins. **B**) Detailed image of the CXCR4 expression in the tumor invasive front, clearly showing a strong CXCR4 expression in the tumor budding, while primary tumor tissue presents a negligible expression of the receptor. Scale bars = 200 µm and 100 µm.

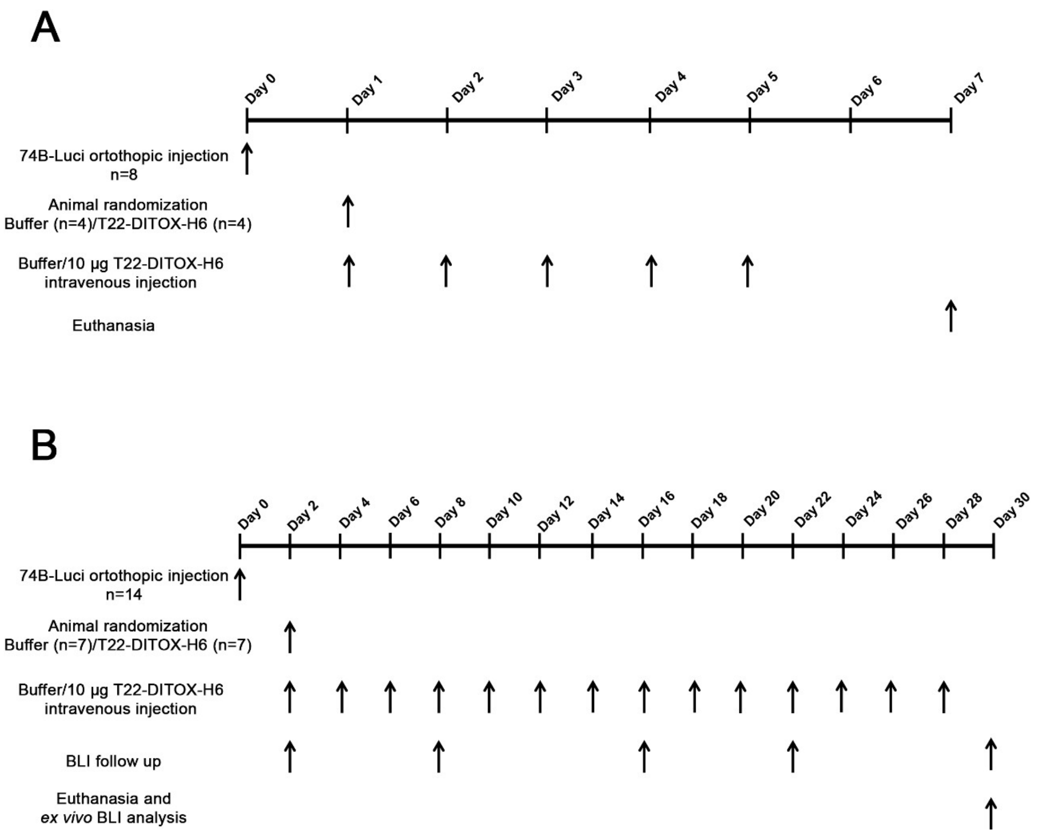

Figure S2. Schematic representation of the experimental design followed in the studies. **A**) Timeline of the experiment that evaluated the T22-DITOX-H6 anti-invasive effect, including all the relevant studies performed. **B**) Experimental design followed during the experiment that assessed the anti-metastatic effect of T22-DITOX-H6 repeated administration, including the study groups, animal number, details on the treatment administration and BLI acquisition, and euthanasia.

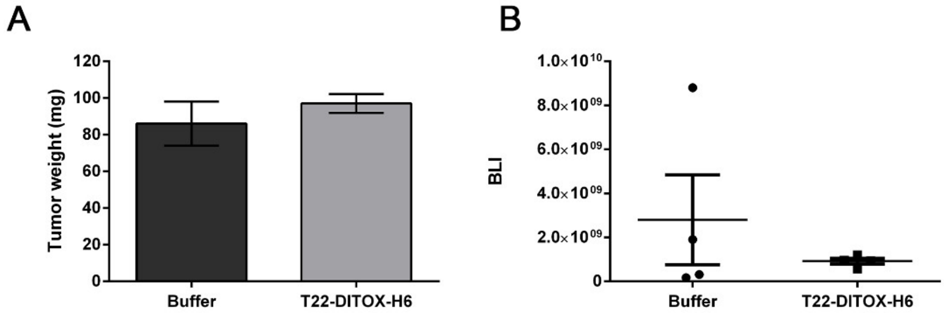

Figure S3. Tumor size assessment in the experiment that evaluated T22-DITOX-H6 anti-invasive effect. **A**) Tumor weight registered at euthanasia for buffer and nanotoxin-treated animals. **B**) BLI emitted by the primary tumors at the end point of the experiment in both buffer and T22-DITOX-H6 groups. No statistical differences were found between groups. n = 4 per group (total animal number 8). Statistical analysis was performed by Mann-Whitney test. Error bars indicate SEM.

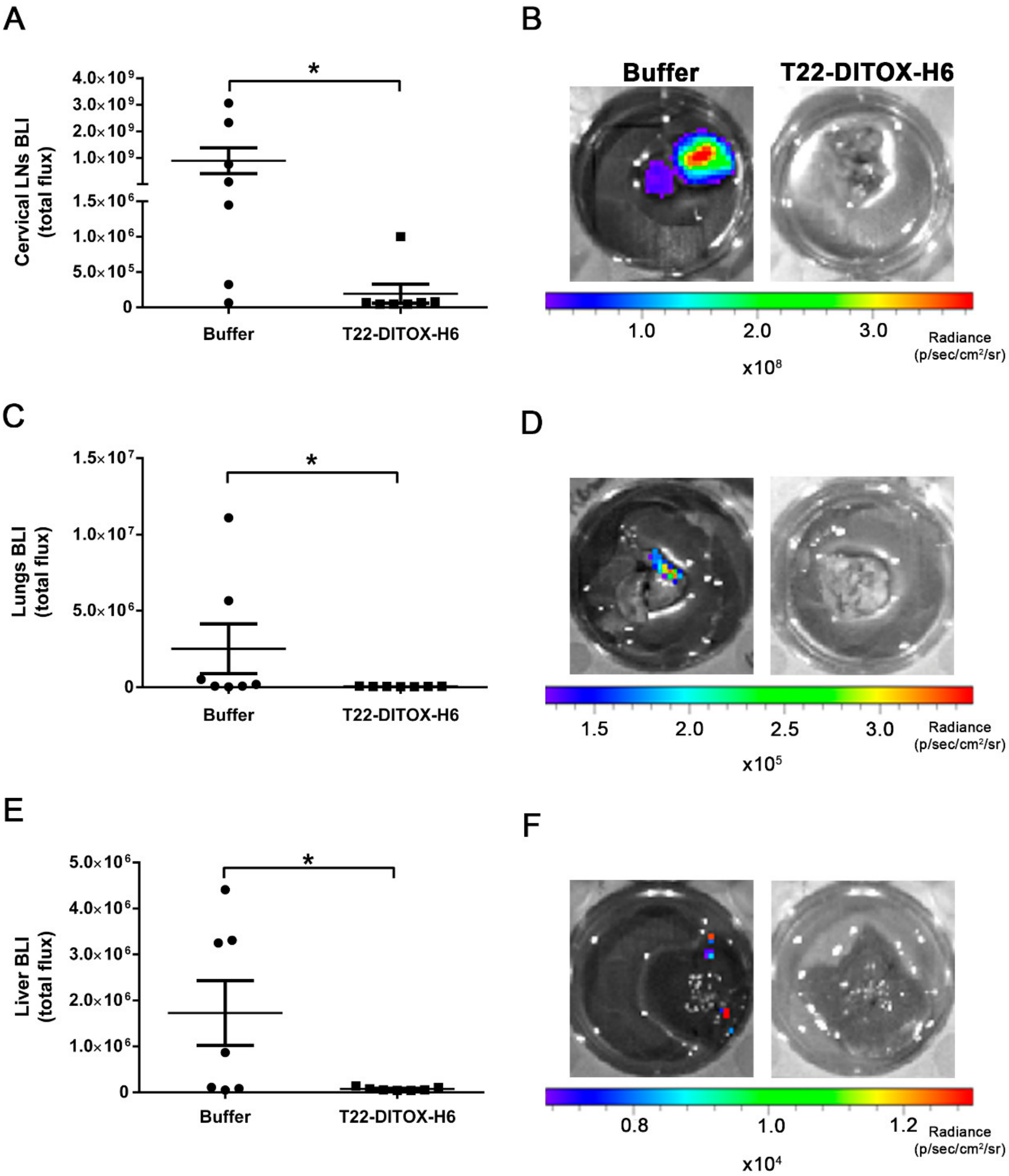

Figure S4. Ex vivo bioluminescence evaluation in the metastatic organs. BLI was semi-quantified in the explanted relevant organs at the endpoint of the experiment that evaluated T22-DITOX-H6 antimetastatic effect. **A**) Semi-quantification of the BLI emitted by the explanted cervical lymph nodes obtained from buffer and nanotoxin-treated animals. **B**) Representative BLI images of cervical lymph nodes from control and T22-DITOX-H6 groups. **C**) Evaluation of the BLI emitted by lung samples from control and T22-DITOX-H6 treated mice. **D**) BLI images of a lung obtained from a control animal and a nanotoxin-treated one. **E**) Assessment of the BLI from livers explanted from buffer and T22-DITOX-H6 groups. **F**) Images showing the BLI emitted by liver samples derived from control and nanotoxin-treated animals. * $p < 0.05$. Statistical analysis was performed by Mann-Whitney test. Error bars indicate SEM.

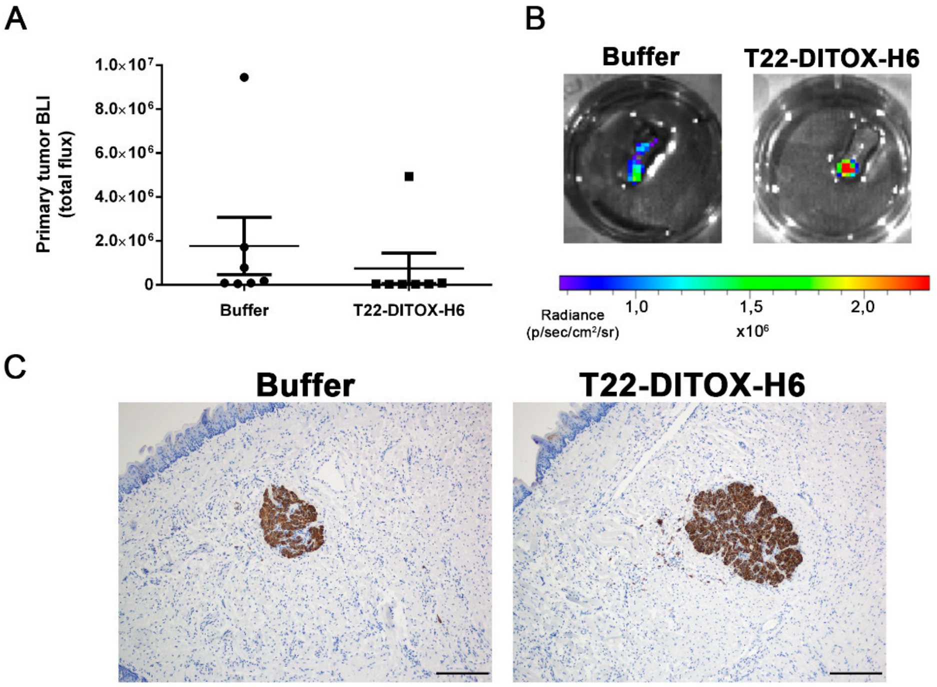

Figure S5. Evaluation of the primary tumor size in buffer and nanotoxin-treated animals in the experiment that evaluated T22-DITOX-H6 antimetastatic effect. **A**) Semi-quantification of the BLI emitted by primary tumors from buffer and nanotoxin-treated animals at the end of the experiment. No statistical differences were found between groups. **B**) Representative BLI images of two primary tumor samples from buffer and T22-DITOX-H6-treated animals. **C**) Anti-Human Vimentin IHC images from buffer and T22-DITOX-H6 primary tumor samples. Scale bar = 200 μm; n = 7 per group (total animal number 14). Statistical analysis performed by Mann-Whitney test. Error bars indicate SEM.
